# Acquisition of innate B cell properties and generation of autoreactive IgA antibodies by follicular B cells during homeostatic proliferation

**DOI:** 10.3389/fimmu.2025.1506628

**Published:** 2025-01-22

**Authors:** Laura Kampe, Christian Melcher, Katrin Westphal, Korbinian Brand, Niko Föger, Kyeong-Hee Lee

**Affiliations:** ^1^ Inflammation Research Group, Hannover Medical School, Hannover, Germany; ^2^ Institute of Clinical Chemistry, Hannover Medical School, Hannover, Germany

**Keywords:** marginal zone B cells, follicular B cells, IgA class-switching, IgA autoantibodies, homeostatic proliferation

## Abstract

The physiologic process of homeostatic proliferation serves to restore the pool of peripheral lymphocytes in response to lymphopenia. However, functional changes in B cell responses during homeostatic proliferation are still only insufficiently characterized. Mature peripheral B cells consist of functionally distinct B cell subsets, such as adaptive follicular B cells (FoBs) and the innate B cell subsets, marginal zone B cells (MZBs) and B1a B cells. During homeostatic proliferation, B cells undergo antigen-independent clonal expansion and differentiation into antibody-producing plasma cells (PCs). However, it is still largely unknown which B cell lineages are involved in the formation of antibodies in response to lymphopenia and what functional properties these antibodies have. Employing adoptive transfer of different mature B cell subsets into lymphopenic *Rag2^-/-^* hosts, we here show that not only innate B cells – MZBs and B1a cells – but also adaptive FoBs were capable to differentiate into PCs and to produce IgM and class-switched IgA serum antibodies in a T cell-independent fashion during homeostatic proliferation. In light of the poor reactivity of FoBs to innate stimulation *in vitro*, the observed high expansion capacity of FoBs, their sustained repopulation of lymphoid and intestinal organs and their particularly prominent ability to induce class-switched auto-/polyreactive IgA antibodies in this antigen- and T cell-independent system was rather unexpected. These properties, which are more typical for innate B cells, were associated with a striking plasticity of FoBs that transdifferentiate into MZB-like cells under lymphopenic conditions. Together, our study indicates that the reconstitution of antibodies in response to lymphopenia-induced homeostatic B cell proliferation is mainly elicited by innate MZB-like B cell responses via antigen- and T cell-independent pathways resulting in the selection of autoreactive IgA antibodies. In addition, our data point to the pathogenic potential of the conversion of conventional adaptive B cells, which are the most common population of mature B cells, into innate-like B cells and the production of autoreactive IgA antibodies during homeostatic proliferation. This process could also manifest as clinical complication of therapy-induced lymphopenia in the context of transplantation and cancer in human patients.

## Introduction

Innate functions of T and B cells, which are classically regarded as antigen-specific adaptive immune cells, have recently gained increased attention as they have opened up new perspectives for a better understanding of the complexity of immune responses. In addition to well-known unconventional innate T cells, such as ILCs and γδ T cells, it is now recognized that there are also functional subsets of B cells, namely marginal zone B cells (MZBs) and B1 B cells which exhibit evolutionarily conserved innate immune functions.

MZB and B1 B cells have innate sensing properties and are highly sensitive in response to Toll-like receptor (TLR) triggering, which contributes to the clearance of pathogens in the blood by T cell-independent antibody responses (1, 2).

The spleen with its highly specialized lymphoid compartments plays an important role for the discrete steps in the final differentiation of B cell subsets. Immature B cells derived from the bone marrow undergo final stages of development to mature B cells in the spleen, either differentiating into naïve follicular B cells (FoBs), or MZB cells, which are named based on their anatomical location. B1 B cells generally arise from fetal tissues and once generated are maintained through self-renewal, although it is also suggested that a small addition of B1 cells can alternatively be developed in the bone marrow during adulthood (3). The spleen is recognized as the most important topographical location for the maintenance of the functional pool of MZB cells (4, 5). Although B1 B cells are predominantly found in the peritoneal and pleural cavities (6), the spleen also plays a crucial role for the generation and retention of the B1a B cell population (7).

Conventional FoBs, which constitute the by far largest population of the peripheral B cell pool, typically participate in T-dependent immune responses to specific foreign antigens and are able to produce high affinity antibodies. In contrast, innate B cell subsets, MZB and B1a B cells, possess an inherent capacity to elicit a rapid, but relatively low affinity T-independent antibody response (1). B1 B cells are considered to be the major source of natural IgM, as well as gut IgA at steady state (8, 9).

Homeostatic proliferation is a physiological process of the immune system to restore and maintain the pool of peripheral lymphocytes in response to lymphopenia (10). Lymphocyte-depleting therapies, such as radiotherapy or the administration of lymphocyte-depleting reagents, which are an increasingly common component during organ and bone marrow transplantations as well as for treatments of cancer or autoimmune disease, lead to lymphopenia-induced homeostatic proliferation of T and B cells (11). Although homeostatic proliferation is an important mechanism for a quantitative return to the equilibrium state of the immune system, there is a growing number of reports suggesting possible deleterious effects. Homeostatic proliferation has been found to be associated with functional alterations of T and B cells, for example in lymphopenia-associated autoimmunity (12, 13) and allograft rejection in transplantation (14–16).

Similar to T cells, the homeostatic proliferation of B cells has been demonstrated in animal models by the transfer of mature B cells into immunodeficient hosts, which leads to the expansion and survival of B cells over longer periods of time through continuous self-renewal processes (17–19). During homeostatic proliferation, naïve B cells show a marked ability to differentiate into IgM-secreting plasma cells in a T cell-independent fashion (17). However, the cellular origin of the antibody producing cell population and the functional properties of the antibodies produced during homeostatic proliferation are not yet fully understood.

## Results

**Figure 1:**
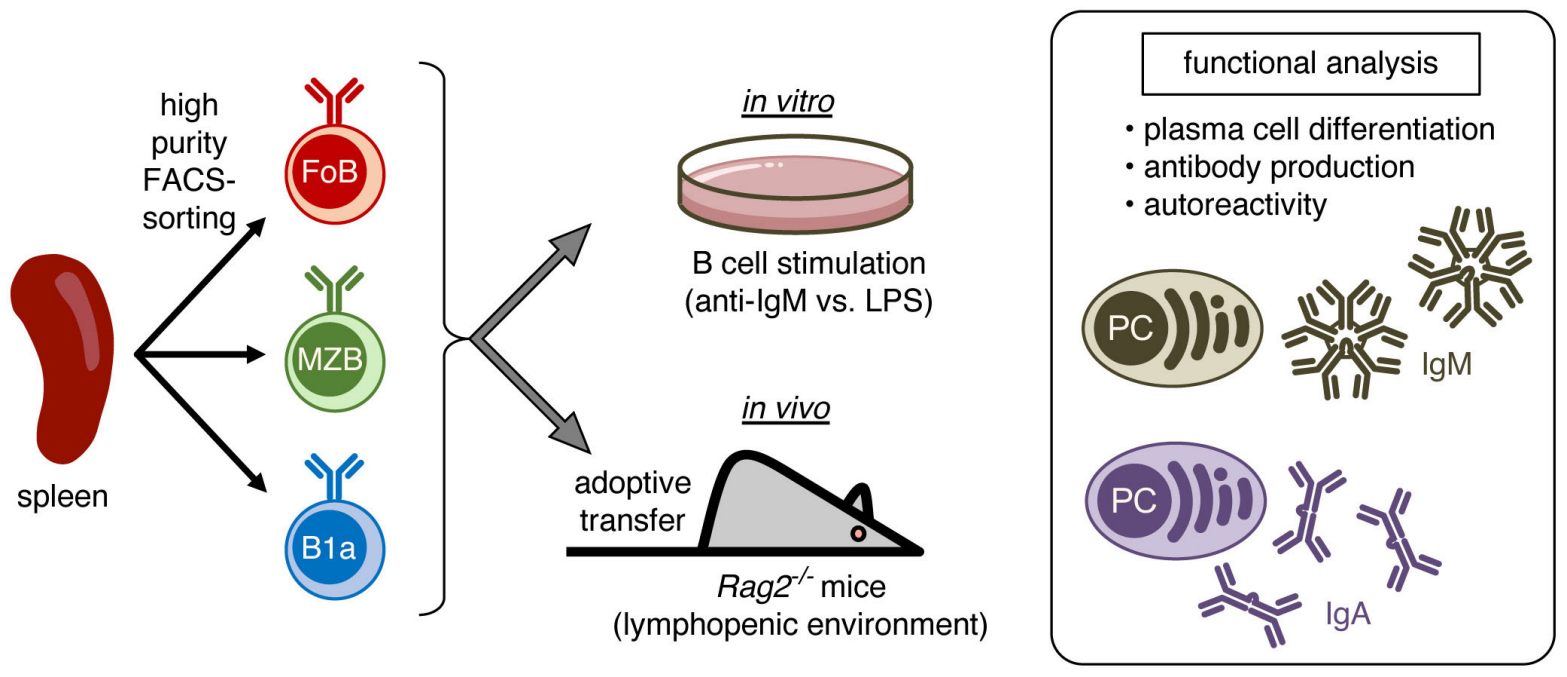
Schematic representation of the experimental approach. Splenic B cells were high purity FACS-sorted into follicular B cells (FoB), marginal zone B cells (MZB), and B1a B cells. Purified B cell subsets were stimulated *in vitro* or were *in vivo* adoptively transferred into lymphopenic *Rag2^-/-^* mice to induce homeostatic proliferation and subsequently analyzed for plasma cell differentiation, production of IgM and IgA antibodies, and antibody autoreactivity.

In this study, we investigated the functional properties of mature B cell subsets in response to T cell-independent lymphopenia-induced homeostatic proliferation. To this end, we performed high purity FACS-sorting of naïve splenic B cells into classical follicular B cells (FoBs), as well as innate marginal zone B cells (MZBs) and B1a cells (**Figure 1**). Purified B cell subsets were then studied in *in vitro* B cell stimulation assays and *in vivo* in the context of homeostatic proliferation by performing adoptive transfer of sorted cells into lymphopenic *Rag2* ^-/-^ hosts. As functional readout, we assessed the capacity of the different B cell subsets to differentiate into plasma cells, to produce IgM and class-switched IgA antibodies and potential autoreactivity of these antibodies.

### Innate B cells are capable of plasma cell differentiation and IgA class-switching in response to TLR4 stimulation

**Figure 2:**
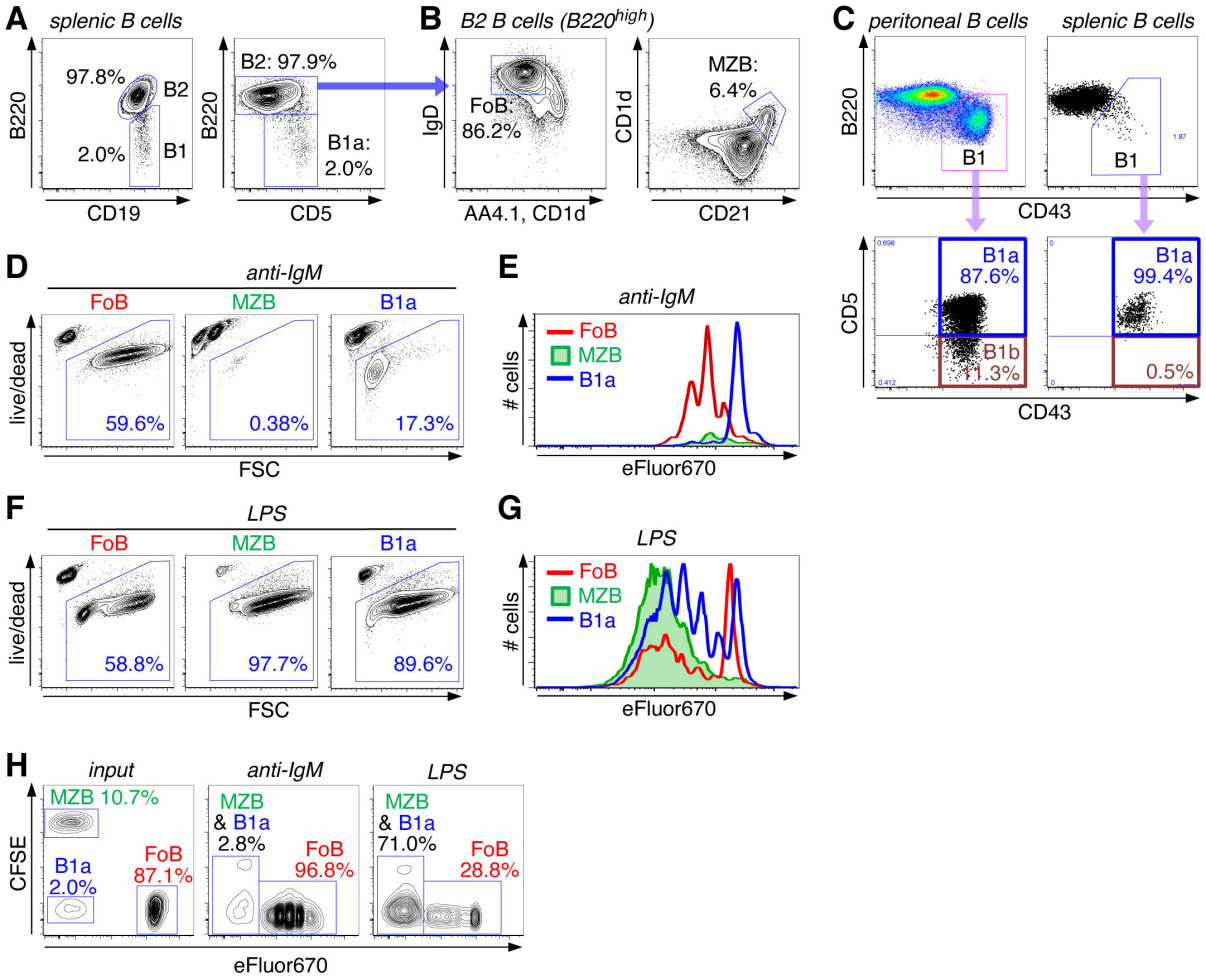
High proliferative capacity of innate B cells upon BCR-independent stimulation via TLR4. **(A, B)** Splenic B cells were stained with the indicated surface markers and analyzed by flow cytometry. **(A)** FACS plots are pregated on live CD45^+^CD19^+^ singlet B cells and show staining for B220^high^ B2 cells and B220^low^ B1 cells (left panel), the latter being classified as B220^low^CD5^+^ B1a cells (right panel). **(B)** FACS plots are gated on CD19^+^B220^high^ B2 cells and show staining for IgD^high^AA4.1^-^CD1d^-^ follicular B cells (FoB) (left panel) and CD1d^high^CD21^high^ marginal zone B cells (MZB) (right panel). **(C)** FACS plots of peritoneal and splenic B cells (pregated on live CD45^+^CD19^+^ singlet B cells) showing staining for B220 and CD43. The B220^low^CD43^+^ B1 cell population was further analyzed for CD43^+^CD5^+^ B1a and CD43^+^CD5^-^ B1b cells. **(D–G)** FACS-sorted splenic B cell subsets (FoB, MZB, and B1a B cells) were stimulated with either anti-IgM **(D, E)** or LPS **(F, G)**. **(D, F)** Cells were analyzed for cell survival versus forward scatter (FSC) (day 3). **(E, G)** Sorted cells were labeled with eFluor670 before addition of the stimulus and then analyzed for cell division (day 3). **(H)** FACS-sorted splenic B cell subsets were differentially labeled with either eFluor670 (FoB), CFSE (MZB) or left unlabeled (B1a). B cell subsets were then mixed as indicated in the input sample (left panel, day 0) in a ratio that is similar to their natural frequencies in the spleen. Cell mixtures were treated with anti-IgM (middle panel) or LPS (right panel) and analyzed by FACS on day 3 of culture. Data are representative of at least 2 independent experiments.

The spleen is the major site for the maintenance of distinct mature B cell subsets, FoBs, MZBs, as well as B1a B cells. These different splenic B cell subsets are characterized by the expression of a combination of specific cell surface markers. Generally, CD19^+^ B1 and B2 B cell subsets are distinguished based on B220 surface expression levels, with B1 cells being B220^low^ and B2 B cells B220^high^ (**Figure 2A**). The B220^high^ B2 B cell population is further subdivided into IgD^high^CD23^high^AA4.1^neg^CD1d^low^CD21^low^ follicular B cells (FoBs) and IgD^low^CD23^low^AA4.1^neg^CD1d^high^CD21^high^ marginal zone B cells (MZBs) (**Figure 2B**). Peritoneal B1 B cells consist of B1a (CD19^+^B220^low^CD43^+^CD5^+^) and B1b B cells (CD19^+^B220^low^CD43^+^CD5^-^), which are distinguished by CD5 expression, while the splenic B1 cell compartment essentially only contains CD19^+^B220^low^CD43^+^CD5^+^ B1a B cells (**Figure 2C**). In our studies, we used the B1a B cell population from the spleen, which contains high numbers of naïve and mature B1a B cells.

Responsiveness of these different types of mature B cells to B cell receptor (BCR) stimulation versus innate triggering via TLRs was assessed by treating highly purified FACS-sorted splenic B cell subsets (FoBs, MZBs and B1a cells) with anti-IgM or the Toll-like receptor 4 (TLR4) ligand LPS (**Figures 2D–G**). Upon anti-IgM stimulation (=BCR triggering), FoBs proliferated vigorously and acquired a blast-like phenotype, as indicated by increased forward scatter (FSC). In contrast, anti-IgM stimulation rather induced cell death in MZBs, while B1a cells were largely unresponsive to this treatment (**Figures 2D, E**). On the other hand, innate MZB and B1a B cell subsets were highly sensitive to LPS stimulation, as revealed by induction of strong proliferation and high cell survival (**Figures 2F, G**). Compared to innate B cell subsets, FoBs were substantially less responsive to LPS treatment.

We further analyzed responsiveness of these different B cell subsets in a more competitive setting that more closely mimics the physiologic situation. Differentially labeled MZBs and FoBs and non-labeled B1a cells were mixed in a ratio that corresponds to the natural frequencies of these major B cell subsets in the spleen (**Figure 2H**, input). Consistent with the high proliferative capacity of FoBs in response to BCR triggering and the high number of FoB input cells, after 3 days of anti-IgM stimulation almost all cells in the culture were of FoB origin and only minimal (<3%) MZB and B1a cells have remained (**Figure 2H**, middle panel). Strikingly, however, despite a rather small percentage of MZB and B1a input cells (in total <13%), MZB- and B1a-derived cells greatly outnumbered FoB-derived cells when the cell mixture was treated with LPS (**Figure 2H**, right panel), highlighting the high proliferative responsiveness of innate B cells to TLR stimulation. These data also indicate that although innate MZB and B1a cells constitute a rather small fraction of splenic B cells, they have the capacity to outperform and even override classical FoBs in the context of an innate, antigen-independent stimulus.

**Figure 3:**
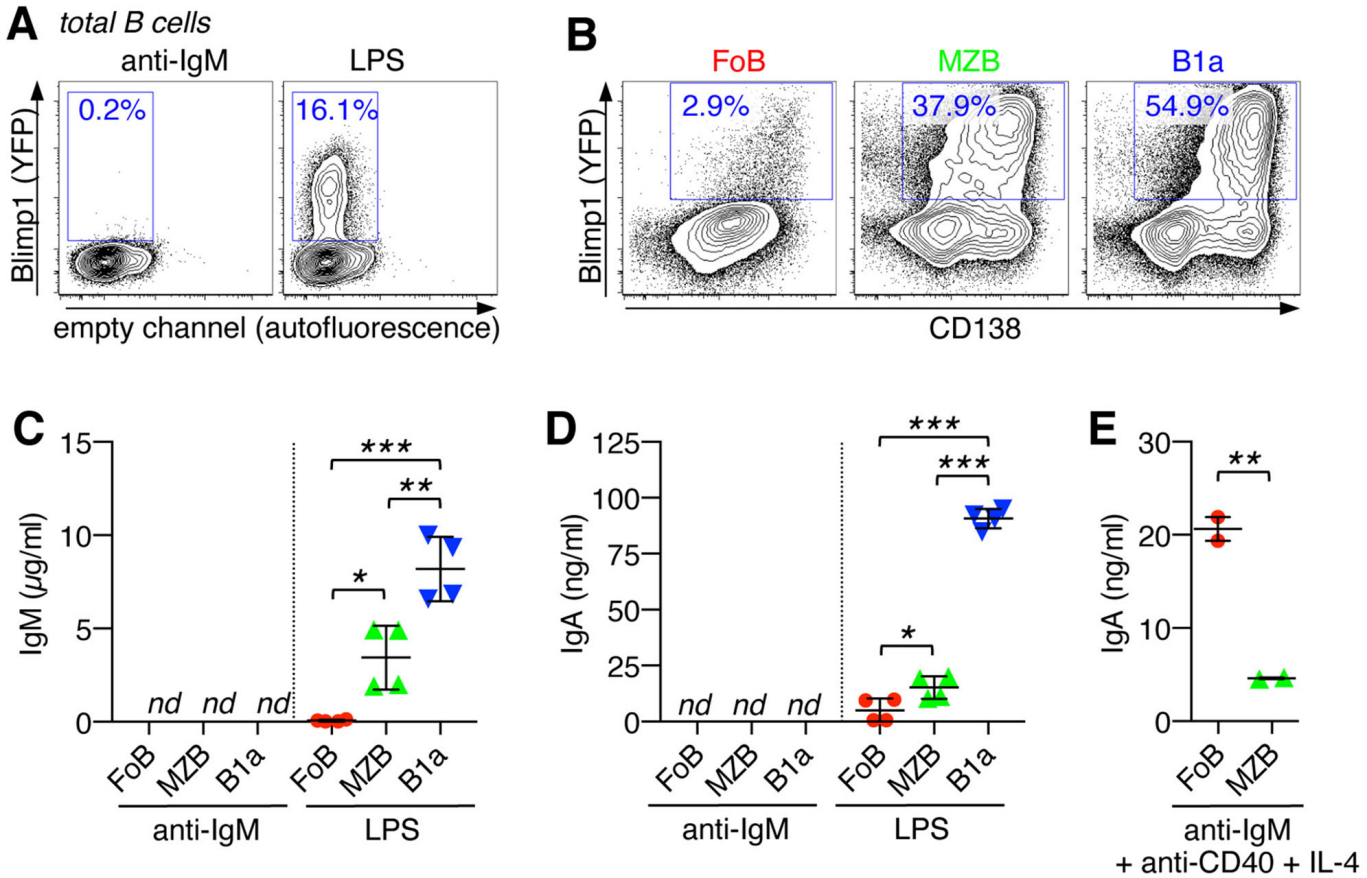
Innate B cells are capable of plasma cell differentiation and IgA class-switching in response to TLR4 stimulation. **(A)** Purified total splenic B cells from Blimp1-YPF reporter mice were stimulated for 3 days with anti-IgM or LPS and analyzed by flow cytometry for the differentiation into Blimp1(YFP)^+^ plasma cells. **(B)** FACS-sorted B cell subsets (FoB, MZB, and B1a B cells) from the spleen of Blimp1-YPF reporter mice were stimulated with LPS and analyzed for the formation of Blimp1(YFP)^+^CD138^+^ plasma cells (day 3). **(C–E)** FACS-sorted B cell subsets (FoB, MZB, and B1a B cells) were stimulated as indicated with LPS (day 3), anti-IgM (day3) or anti-IgM + anti-CD40 + IL-4 (day 6) and culture supernatants were analyzed by ELISA for **(C)** IgM and **(D, E)** IgA antibody levels. Lines indicate mean ± SEM; *nd*, not detectable; *p < 0.5; **p < 0.01; ***p < 0.001; one-way ANOVA and Tukey’s multiple comparisons test. Data are representative of at least 3 independent experiments.

To study plasma cell (PC) differentiation upon activation of naïve B cells, we employed the Blimp1-YPF reporter mouse system, in which yellow fluorescent protein (YFP) is under control of the Blimp1 promoter (20) and expression of YFP thus restricted to PCs. Upon activation with the TLR4 ligand, LPS, purified total splenic B cells from naïve Blimp1-YPF mice efficiently induced formation of Blimp1(YFP)^+^ PCs within 3 days, however, in response to B cell receptor (BCR) stimulation via anti-IgM treatment hardly any Blimp1(YFP)^+^ PCs could be detected (**Figure 3A**).

Further analysis using highly purified FACS-sorted splenic B cell subsets, FoBs, MZBs and B1a cells, revealed that LPS-induced PC differentiation mainly originated from MZB and B1a B cell populations, which is consistent with the high responsiveness of these cells to TLR stimulation. Upon LPS stimulation, >54% of B1a cells and >37% of MZBs had differentiated into Blimp1(YFP)^+^/CD138*^+^* PCs, while PC formation was barely detectable in LPS-treated FoBs (**Figure 3B**).

Our analysis further evaluated the ability of MZB- and B1a B cell-derived PCs to produce antibodies upon innate triggering. After 3 days of LPS-treatment, antibody production was barely detectable in cultures of FoBs, which is in line with minimal PC formation by FoBs under this innate stimulation condition (**Figures 3C, D**). Consistent with poor PC differentiation upon BCR triggering, culture supernatants from anti-IgM-treated B cell subsets had non-detectable levels of IgM and IgA antibodies (**Figures 3C, D**). However, IgM and, importantly, although to a relatively low degree, also IgA antibodies were found in cultures of LPS-treated MZBs and B1a B cells, with B1a cell culture supernatants showing by far the highest IgM and IgA antibody levels (**Figures 3C, D**). Flow cytometric analysis further demonstrated the ability of MZBs and B1a cells to rapidly generate IgA class-switched Blimp1(YFP)^+^ PCs upon days of LPS stimulation (**Supplementary Figure S1A**). Upon LPS-treatment for longer periods of time (day 7), IgA^+^ and IgG^+^ Blimp1^+^ PCs were also detectable in cultures of FoBs, indicating that FoBs are principally capable to differentiate into IgA- (and IgG)-producing PCs in response to TLR4, but are less efficient and exhibit slower kinetics as innate MZBs and B1a cells (**Supplementary Figure S1B**). Note that at this later time point cultures of MZBs and B1a cells were already largely collapsed due to cellular overactivation.

Although FoBs were less efficient to produce class-switched IgA antibodies in response to LPS, FoBs exhibited robust differentiation into IgA- (and IgG)-producing PCs after 6 days of stimulation with anti-IgM + anti-CD40 + IL-4 that mimics germinal center (GC) responses (**Figure 3E** and **Supplementary Figure S1B**). IgA production was substantially lower in MZBs when BCR engagement was combined with anti-CD40 and IL-4 (**Figure 3E**), suggesting that BCR-induced cell death is dominant over CD40 signaling in MZBs.

Taken together, our data reveal different IgA class-switching capacities of adaptive FoBs versus innate MZB and B1a cells. FoBs undergo PC differentiation and IgA class switching through BCR- and GC-dependent pathways, whereas innate MZB and B1a B cells rapidly differentiate into PCs and undergo IgA-class switching under BCR-independent innate stimulation conditions.

### Transdifferentiation of FoBs into MZB-like innate B cells and IgM PC differentiation of naïve B cell subsets under lymphopenic conditions in the spleen

**Figure 4:**
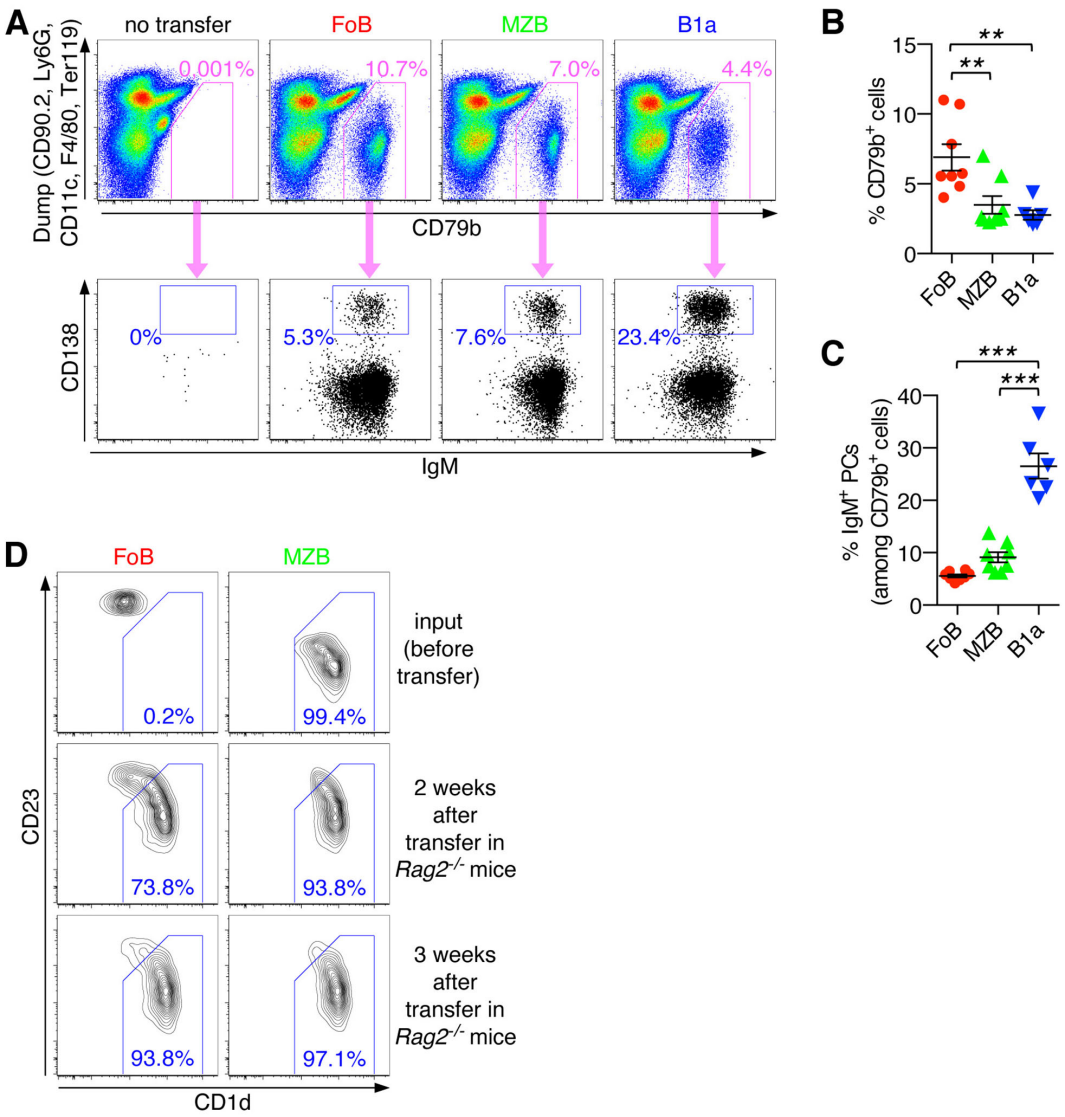
Transdifferentiation of FoBs into MZB-like innate B cells and IgM PC differentiation of naïve B cell subsets under lymphopenic conditions in the spleen. **(A-C)** Follicular B cells (FoB) and marginal zone B cells (MZB) were purified by fluorescence-activated cell sorting and adoptively transferred into *Rag2^-/-^* mice. After 3 weeks, spleens of recipient mice that had received the indicated B cell subsets were analyzed for the presence of CD79b^+^ B cells and IgM^+^ plasma cells (PCs). **(A)** Representative FACS plots are pregated on live CD45^+^ singlet immune cells and show staining for CD79b against a ‘dump’ channel to identify the CD79b^+^ B cell population (upper panel) which is then further analyzed for CD138^+^IgM^+^ plasma cells (PCs) (lower panel). **(B)** Frequency of CD79b^+^ cells (among the CD45^+^ immune cell population). **(C)** Frequency of IgM^+^ PCs (among CD79b^+^ cells). Each symbol represents an individual mouse; lines indicate mean ± SEM; **p < 0.01; ***p < 0.001; one-way ANOVA and Tukey’s multiple comparisons test. **(D)** FoB, MZB, and B1a B cells were purified by fluorescence-activated cell sorting and adoptively transferred into *Rag2^-/-^* mice. At the indicated times spleens of recipient mice were subjected to flow cytometric analysis. FACS plots are gated on live CD45^+^CD79b^+^ singlet B cells and show staining for CD1d vs CD23 on input cells (sorted cells before transfer) and on splenic B cells at week 2 and 3 after transfer into *Rag2^-/-^* mice. Numbers indicate the frequency of cells with a CD1d^high^CD23^low^ marginal zone B cell-like phenotype. Data are representative of at least 3 independent experiments.

We next investigated the functional activities of different mature B cell subsets in response to lymphopenia-induced homeostatic proliferation. To this end, we employed adoptive transfer of highly purified FACS-sorted FoBs, MZBs and B1a B cells into *Rag2* ^-/-^ mice that lack mature B and T cells. Descendants of all three adoptively transferred B cell subsets (FoBs, MZBs, B1a B cells) could readily be recovered from the spleen, with FoBs showing significantly higher capacity to colonize the spleen compared to MZB and B1a cells, (**Figures 4A, B**, and **Supplementary Figure S2**). Notably, we observed that under these lymphopenic conditions FoBs gradually acquire a MZB-like phenotype. 2-3 weeks after transfer the majority of FoBs had downregulated expression of CD23 and upregulated expression of the typical MZB surface marker CD1d to similar levels as observed in MZBs (**Figure 4D**). This ability of FoBs to transdifferentiate into MZBs in a lymphopenic environment is consistent with previous work showing that homeostatic proliferation promotes functional plasticity and induction of a MZB gene signature in FoBs (21). Transdifferentiation to MZBs was not observed for B1a B cells (**Supplementary Figure S3**). Given that FoBs are the most abundant type of mature B cells, our data suggest that B cells generated by lymphopenia-induced homeostatic proliferation represent mainly transdifferentiated MZB-like innate B cells.

Our analysis further showed that all three adoptively transferred B cell subsets (FoBs, MZBs, B1a B cells) exhibited the ability to differentiate into IgM-producing PCs. A substantial proportion of FoBs and MZBs were found to have differentiated into CD138^+^IgM^+^ PCs in the spleen (5.5 ± 0.3% and 9.1 ± 0.9%, respectively). The fraction of splenic IgM^+^ PCs was, however, clearly highest in the B1a B cell population (26.5 ± 2.4%) (**Figures 4A, C**). Note that a high efficiency of B1a cells to differentiate into IgM-producing PCs was similarly observed in the *in vitro* system and may be generally related to the high ability of B1a B cells to produce natural IgM under physiological conditions. Furthermore, while under *in vitro* culture conditions FoBs showed only minimal PC differentiation upon T-independent stimulation, FoBs exhibited formation of IgM^+^ PCs with similar efficiency as MZBs in the *in vivo* adoptive transfer model, which likely is related to the observed transdifferentiation of FoBs to MZB-like cells in this system of lymphopenia-induced homeostatic proliferation.

Notably, splenic CD138^+^ PCs were almost exclusively of the IgM-type (**Figure 4A**, lower panel), suggesting that the spleen is a main site for the formation of IgM-producing PCs in response to homeostatic B cell proliferation.

### Capacity of naïve B cell subsets to differentiate into class-switched IgA^+^ PCs in intestinal organs under lymphopenic conditions

**Figure 5:**
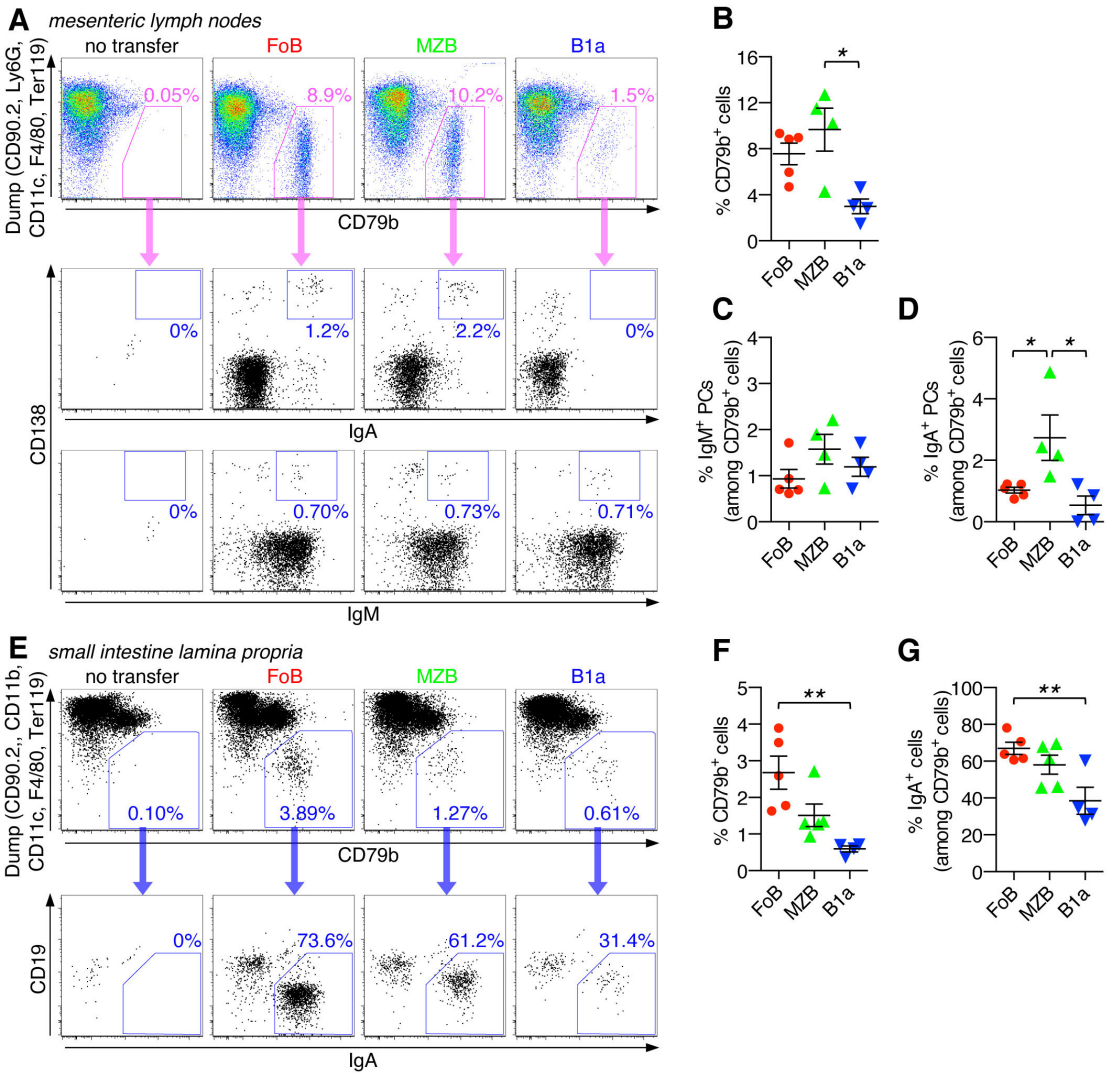
Capacity of naïve B cell subsets to differentiate into class-switched IgA^+^ PCs in intestinal organs under lymphopenic conditions. Follicular B cells (FoB), marginal zone B cells (MZB), and B1a B cells were purified by fluorescence-activated cell sorting and adoptively transferred into *Rag2^-/-^* mice. After 3 weeks, mice that had received the indicated B cell subsets were analyzed for the presence of CD79b^+^ B cells and their differentiation into IgA^+^ and/or IgM^+^ plasma cells (PCs) in mesenteric lymph nodes **(A-D)** and small intestine lamina propria **(E-G)**. **(A, E)** FACS plots are pregated on live CD45^+^ singlet immune cells and show staining for CD79b against a ‘dump’ channel to identify the CD79b^+^ B cell population (upper panel) which is then further analyzed for IgA^+^ and/or IgM^+^ plasma cells (lower panel(s)). **(B, F)** Frequency of CD79b^+^ cells (among the CD45^+^ immune cell population). Frequency of **(C)** IgM^+^ or **(D, G)** IgA^+^ PCs (among CD79b^+^ cells). Each symbol represents an individual mouse; lines indicate mean ± SEM; *nd*, not detectable; *p < 0.05; **p < 0.01; one-way ANOVA and Tukey’s multiple comparisons test. Data are representative of at least 3 independent experiments.

To further assess the capacity of innate B cells to differentiate into PCs and to undergo antibody class-switching, we extended our analysis to peripheral lymph nodes and intestinal organs of *Rag2^-/-^* mice that had received adoptively transferred naïve FoBs, MZBs, and B1a B cells. All transferred B cell subsets were able to colonize mesenteric lymph nodes (mLNs), with lowest efficiency being observed for B1a cells (**Figures 5A, B**, and **Supplementary Figure S2**). Low frequencies of IgM^+^ PCs were detected for all transferred B cell subsets in mLNs (**Figure 5C**). However, for FoB-derived, and even more so for MZB-derived cells, a noticeable fraction of IgA^+^ PCs could also be detected in mLNs, whereas the percentage of IgA^+^ PCs was lower for B1a-derived cells (**Figures 5A, D**).

Descendants of all adoptively transferred B cell subsets could also be detected in the small intestine, with FoB-derived cells showing the relatively highest colonizing capacity for this organ (**Figures 5E, F**, and **Supplementary Figure S2**). Importantly, in the small intestine, a large fraction of recovered B cells were PCs of the IgA-type, with the percentage of IgA^+^ PCs being highest for FoB-derived cells (67.0 ± 3.2%), followed by MZB-derived cells (58.1 ± 5.1%) and B1a-derived cells (38.5 ± 7.3%) (**Figure 5G**). Our data thus indicate that FoBs are the major source for IgA^+^ PCs in intestinal tissue upon B cell transfer into *Rag2^-/-^* mice. Given the low responsiveness of FoBs to T-independent innate stimuli *in vitro*, the observed highly efficient formation of IgA^+^ PCs by FoB-derived cells upon transfer in a lymphopenic host was rather unexpected, but may be functionally linked to the transdifferentiation of FoBs that acquire a MZB-like phenotype (**Figure 4C**). Considering the transdifferentiation of FoBs to MZB-like cells under lymphopenic conditions, it is currently unclear whether in this context IgA antibodies produced by PCs that have originated from FoBs also reflect typical properties of MZB-derived antibodies.

### Autoreactive IgA antibody production upon adoptive transfer of naïve B cell subsets into lymphopenic *Rag2^-/-^* mice

**Figure 6:**
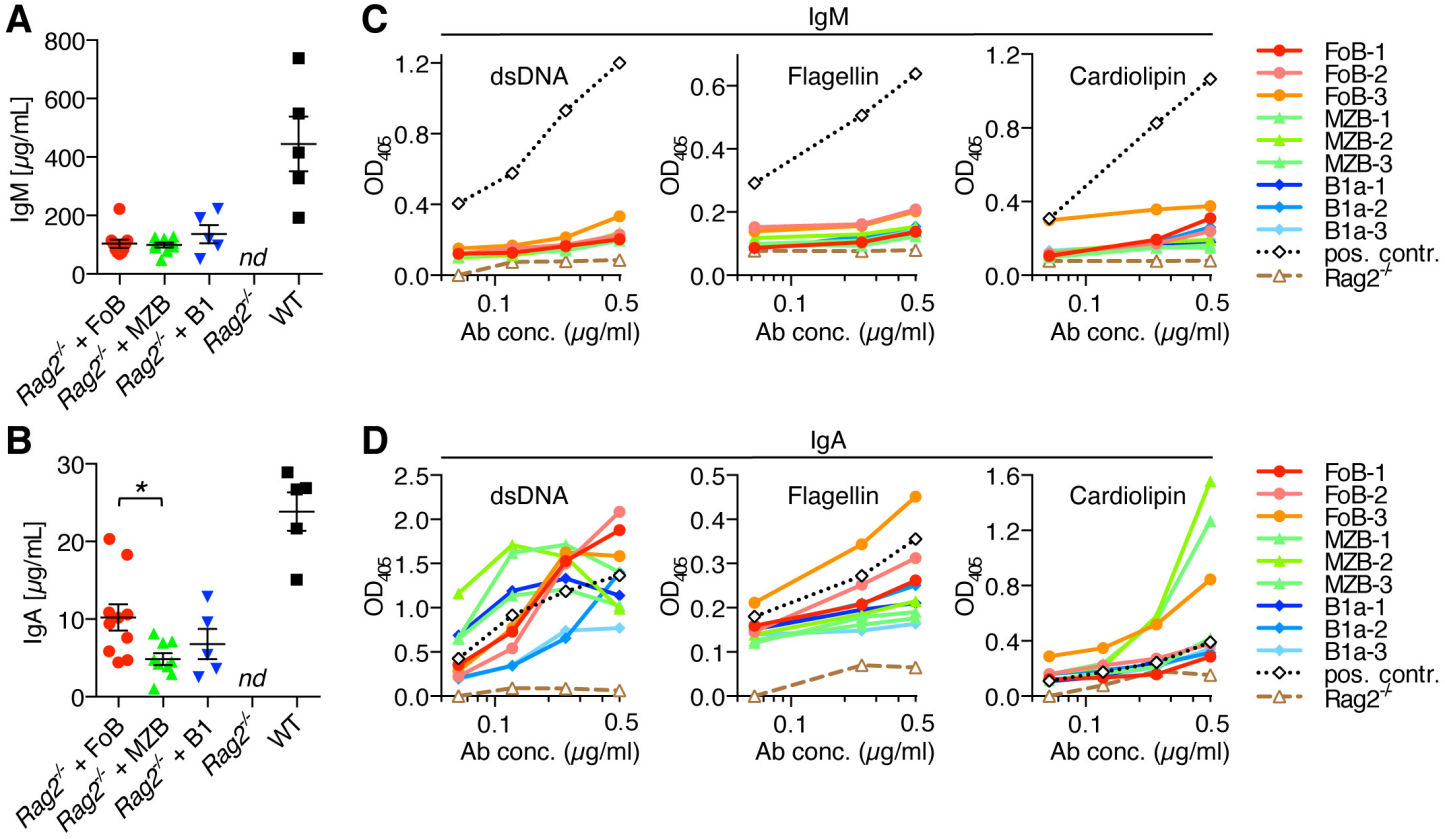
Autoreactive IgA antibody production upon adoptive transfer of naïve B cell subsets into lymphopenic *Rag2^-/-^* mice. Follicular B cells (FoB), marginal zone B cells (MZB), and B1a B cells were purified by fluorescence-activated cell sorting and adoptively transferred into *Rag2^-/-^* mice. **(A)** IgM and **(B)** IgA antibody levels in the serum of *Rag2^-/-^* mice that had received the indicated B cell subsets or from *Rag2^-/-^* control mice in comparison to serum from normal wild type (WT) mice. Each symbol represents an individual mouse; lines indicate mean ± SEM; *nd*, not detectable; *p < 0.05; one-way ANOVA and Tukey’s multiple comparisons test. **(C, D)** ELISA measuring the polyreactivity of **(C)** IgM antibodies and **(D)** IgA antibodies from the serum of *Rag2^-/-^* mice that had received adoptive transfer of FoB, MZB or B1a B cells. Assays were performed with normalized concentrations of IgM and IgA serum antibodies (0.5 µg/ml and three additional 1:2 dilutions). Data show the reactivity against dsDNA, flagellin and cardiolipin. Dotted lines (black color) represent a positive control serum from an aged *Fcmr^-/-^* mouse which has high levels of auto-/polyreactive antibodies (22–24). Dashed lines (brown color) show negative control serum form normal *Rag2^-/-^* mice. Each line represents an individual mouse. Data are representative of at least 3 independent experiments.

We thus further evaluated the functional characteristics of antibodies generated by transferred B cell subsets in lymphopenic *Rag2^-/-^* mice. Analysis of serum antibody levels showed that all *Rag2^-/-^* mice that had received adoptively transferred FoBs, MZBs or B1a B cells had substantial levels of IgM and also class-switched IgA in their serum, in contrast to antibody-deficient *Rag2^-/-^* control mice (**Figures 6A, B**). When compared to serum from normal wild type mice, induction of IgG serum levels by transferred B cell subsets was rather low (**Supplementary Figure S4**). For serum IgM, concentrations of more than 100 µg/ml were detected on average in recipient mice, with similar levels being induced by all three transferred B cell subsets (**Figure 6A**). Although B1a cells consistently showed the lowest recovery rates in all organs examined, IgM antibody production from this innate B cell subset seemed to be compensated by their high cell-intrinsic capacity to differentiate into IgM-producing PCs in the spleen. FoBs and MZBs, on the other hand, showed significantly lower potential to generate splenic IgM^+^ PCs in the *Rag2^-/-^* transfer system, but were generally present in higher numbers compared to B1a B cells, overall resulting in comparable IgM serum levels for all three transferred B cell subsets.

Adoptive transfer of any of the three B cell subsets into *Rag2^-/-^* mice not only resulted in the production of IgM antibodies, but also in the generation of considerable amounts of serum IgA. Notably, serum IgA concentration was highest in *Rag2^-/-^* recipient mice that had received FoBs (12.8 ± 2.1 µg/ml), as compared to MZBs (5.9 ± 0.6 µg/ml) and B1a B cells (6.8 ± 1.9 µg/ml) (**Figure 6B**). This finding is consistent with the high recovery rate of FoB-derived cells in recipient mice and the high frequency of intestinal IgA^+^ PCs in FoB-transferred mice.

Given the ability of naïve B cell subsets to generate both serum IgM and IgA antibodies in the context of homeostatic proliferation, we next investigated the potential auto-/polyreactivity of these antibodies. Our analysis revealed that - compared to serum from *Fcmr^-/-^* mice that are known to have increased levels of natural autoantibodies (22–24) - IgM serum antibodies that were produced upon adoptive transfer of naïve B cell subsets into *Rag2^-/-^* mice showed only low reactivity towards double-stranded DNA (dsDNA), flagellin and cardiolipin. However, IgA serum antibodies from these mice strikingly exhibited broad and strong reactivity against these structurally diverse antigens (**Figures 6C, D**). In particular, serum IgA antibodies from FoB-transferred mice showed high binding to dsDNA and flagellin, while IgA from MZB-transferred mice showed highest reactivity to the mitochondrial membrane lipid component cardiolipin. Although within the relatively short 3-week time frame of the experiments animals did not develop any overt signs of disease, it is worth mentioning that both anti-dsDNA and anti-cardiolipin antibodies occur in various autoimmune disorders, especially in patients with systemic lupus erythematosus (SLE), suggesting potential pathogenic autoactivity of these antibodies. B1a-derived IgA antibodies also exhibited binding to the tested antigens, particularly to dsDNA, although overall the reactivity of B1a-derived antibodies was lowest among the three B cell subsets. Our data on the production of high levels of auto-/polyreactive IgA antibodies in response to lymphopenia-induced homeostatic B cell proliferation, particularly produced by FoB- and MZB-derived PCs, suggest that potential pathological effects could be conceivable for therapy-induced lymphopenia in humans. Production of auto-/polyreactive antibodies by FoB-derived cells is noteworthy, as, in contrast to the known polyreactivity of innate B cells (MZB and B1a B cells), FoBs are typically considered to be largely non-self-reactive. Transdifferentiation of FoBs to MZB-like cells during homeostatic proliferation, thus, seems to be associated with a skewing of the BCR-repertoire towards higher self-reactivity.

## Discussion

Adoptive transfer of mature B cells into lymphopenic *Rag2^-/-^* hosts leads to the homeostatic proliferation of B cells that is mediated by T cell-independent mechanisms in response to a lymphopenic environment.

It is well recognized that FoBs generally generate high-affinity antibodies against specific antigens via T cell-dependent mechanisms in germinal center reactions (25). Therefore, our finding of efficient formation of IgM and IgA PCs during T cell-independent homeostatic proliferation was rather unexpected. FoBs transferred into lymphopenic *Rag2^-/-^* hosts exhibited highest expansion of IgA-switched PCs in intestinal organs and also produced the highest amounts of serum IgA antibodies compared to innate MZB and B1a B cell subsets. Also, most noteworthy, in agreement with a previous report (21), our data validated the specific plasticity of FoBs that acquire a MZB-like phenotype during homeostatic proliferation. We suggest that the unusual T-independent IgA class-switched antibody response of FoBs is related to the transdifferentiation of FoBs into MZB-like cells in the lymphopenic environment. This process of transdifferentiation has been described to be mediated by sustained activation of Notch signaling during lymphopenic conditions in the spleen (21). Notch signal-dependent lineage conversion from FoBs to MZB cells has also been described in immunocompetent wild type mice (26). In addition, lymphopenia in Rag-mutant mice and patients is associated with increased levels of B cell-activating factor (BAFF) and BAFF signaling has been demonstrated to play a critical role in the survival and proliferation of transferred splenic B cells in *Rag* ^-/-^ hosts (27). The strong expansion of FoB-derived cells in the spleen and intestinal organs of *Rag2^-/-^* hosts and their ability to produce high levels of IgM and class-switched IgA antibodies in a T-independent fashion is thus dependent on BAFF-mediated pro-survival signals and likely associated with the Notch-mediated process of transdifferentiation that may provide additional long-term activatory signals and confers innate-like properties to the converted cells. On the other hand, the relatively low responsiveness of B1a cells to BAFF signals may underlie their lower *in vivo* frequencies compared to FoBs and MZBs, although B1a cells still efficiently differentiated into antibody-producing PCs.

An important but largely unexplored aspect is, however, the BCR-specificity of MZB-like cells that have transdifferentiated from FoBs. We here show that in the specific context of a lymphopenic environment, FoBs generated poly-/autoreactive IgA antibodies, although FoBs typically generate high affinity monoreactive antibodies that normally are not self-reactive. Antibodies induced by FoBs during homeostatic proliferation more resembled MZB-derived antibodies, which are known to have auto-/polyreactive binding characteristics (28, 29). The mechanism and potential selection processes of how FoBs obtain autoreactivity during transdifferentiation are currently unknown.

A causative role of lymphopenia-induced proliferation has been demonstrated for the development of certain autoimmune disorders in animal models (30, 31) and a strong correlation between lymphopenia and autoimmunity has also long been observed in humans. Lymphopenia is for example frequent in patients with SLE in which multiple autoantibodies are associated with lymphopenia (32). It is thus conceivable that autoreactive, class-switched IgA antibodies could also be increasingly selected in humans under therapy-related lymphopenia conditions. This notion is supported by previous reports showing pathogenic B cells in chronic graft-versus-host disease, a severe complication of allogeneic bone marrow transplantation in which B cell autoreactivity and antibody production play an important pathogenic role in human patients (15, 16).

Given that FoBs are the most abundant population of peripheral mature B cells, our data therefore suggest that pathological effects of transdifferentiation of adaptive FoBs into innate-like B cells during homeostatic proliferation should be considered in clinical settings as this could be highly relevant for lymphocyte depletion therapies in the context of transplantation and treatment of autoimmune diseases and cancer.

## Materials and methods

### Mice

Blimp1-YFP reporter mice (B6.Cg-Tg(Prdm1-EYFP^1Mnz^/J); RRID: IMSR_JAX:008828), Rag2^-/-^ mice (B6.Cg-*Rag2^tm1.1Cgn^*/J; RRID: IMSR_JAX:008449) and C57BL/6J (RRID: IMSR_JAX:000664) were originally obtained from The Jackson Laboratory. Mice with deletion of Fcmr (also known as Faim3 or Toso) (*Faim3^tm1.2Khl^*) generated on C57BL/6 background have been described previously (22). All mouse strains utilized in the studies were on C57BL/6J background. Following entry of the mice into the animal facility via embryo transfer, mice were maintained in individually ventilated cages (IVC) under specific pathogen-free (SPF) conditions in the barrier facility at Hannover Medical School. In all experiments age and gender matched 10- to 12-weeks old mice were used. Animal experiments were performed in accordance with institutional guidelines and were approved by the local authorities (Lower Saxony State Office for Consumer Protection and Food Safety, Germany).

### *In vitro* B cell stimulation and cell sorting

For the *in vitro* analysis of B cell proliferation and plasma cell differentiation, untouched murine B cells from C57BL/6J or Blimp1-YFP reporter mice were isolated using Pan B cell isolation kit (Miltenyi Biotec) and either used directly or different B cell subsets were further purified by FACS-sorting (FACSAria Fusion, BD Biosciences). Purified B cell populations were cultured with either LPS (500 ng/ml, InvivoGen)), anti-IgM (20 µg/ml, goat-anti-mouse F(ab’)2 fragment, Jackson ImmunoResearch), or anti-IgM + anti-CD40 (10 µg/ml; clone 1C10, Biolegend) + IL-4 (100 ng/ml; eBioscience). To assess B cell proliferation, purified B cells were labeled with eFluor670 or CFSE, stimulated with anti-IgM or LPS and analyzed for cell division by flow cytometric measurement of eFluor670 or CFSE dilution.

### Adoptive transfer of B cell subsets

Highly purified splenic FoB cells (CD19^+^B220^+^IgD^high^AA4.1^-^CD1d^-^), MZBs (CD19^+^B220^+^AA4.1^-^CD21^+^CD1d^+^), and B1a B cells (CD19^+^B220^low^AA4.1^-^CD1d^-^CD5^+^) from C57BL/6J, were obtained by two-step purification; first total B cell isolation using the Pan B cell negative isolation kit (Miltenyi Biotec) and then further FACS-sorting using a 4-laser FACSAria II/Fusion (BD Biosciences). Sorted B cell subsets were resuspended in sterile PBS, and *i.v.* injected into *Rag2^–/–^* recipients (3×10^6^ to 4×10^6^ cells/recipient). Three weeks after adoptive transfer, cells were isolated from the spleen, mLNs and lamina propria cells from the small intestine of recipients and analyzed by flow cytometry for B cell markers and IgM or IgA PC differentiation.

### Isolation of lamina propria cells from the small intestine

Single cell suspensions were prepared from the small intestine by a combination of mechanical dissociation and enzymatic degradation using the gentleMACS™ Dissociator and the Lamina Propria Dissociation Kit (Miltenyi Biotec GmbH, Germany). Briefly, intestinal tissue was cleared of contents, washed and fat tissue was removed. Intestinal tissues were then cut into small pieces and treated twice for 15 min with predigestion solution (5 mM EDTA, 5% FBS, 1 mM DTT in HBSS w/o Ca^++^ and Mg^++^) at 37°C under shaking. Following incubation with HBSS for 15 min at 37°C, tissue was transferred into gentleMACS C tubes containing the enzyme mix provided by the Kit and incubated for 20 min at 37°C under shaking. Tubes were loaded onto the gentleMACS™ Dissociator for further enzymatic digestion and mechanical disruption using the run program 37C_m_LPDK_1. Samples were then collected by centrifugation, washed twice with cold medium (RPMI, 10% FBS) and processed immediately for further applications.

### Flow cytometry

Single-cell suspensions of spleen, mesenteric lymph nodes, and lamina propria cells from the small intestine were isolated from fresh tissue using standard procedures. Following red blood cell lysis, cells were blocked with anti-CD16/32 (clone 93, Biolegend) and rat serum (Biolegend) and subsequently stained with fluorescent-labeled anti-mouse mAbs: anti-CD45 (clone A20), CD19 (clone 6D5), B220 (clone RA3-6B2), CD43 (clone S11), CD79b (clone HM79-12), CD93 (clone AA4.1), CD1d (clone 1B1), CD21 (clone 7E9), CD23 (clone B2B4), IgD (clone 11-26c.2a), IgM (clone RMM-1), IgG (poly4053), CD5 (clone 53-7.3), CD138 (clone 281-2), Ter119 (clone TER-119) all mAbs from BioLegend, and anti IgA (clone mA-6E1, eBioscience). For live/dead cell discrimination, we used the fixable viability dye eFluor780 (eBiosciences). Flow cytometric measurements were performed on a FACSCantoII cell analyzer (BD Biosciences). Data were analyzed with FlowJo software (version 9.9.6, Mac; BD).

### Polyreactivity/autoreactivity ELISA

Serum titers of IgM, IgG and IgA were determined by specific ELISA kits (eBioscience) according to the manufacturer’s protocol. Polyreactivity ELISA assays were performed similarly as previously described (33). Briefly, high-binding ELISA plates were coated overnight with double-stranded (ds) DNA from calf thymus (10 µg/ml) or flagellin (2 µg/ml) diluted in carbonate buffer. For cardiolipin ELISAs, plates were coated with cardiolipin (10 µg/ml diluted in 100% ethanol) and left uncovered overnight to allow evaporation. The plates were washed 4 times with H_2_Odd, and then blocked with 1 mM EDTA (in TBST) for 1 h at 37°C. After washing the plates 4 times with water, serum IgM and IgA diluted in TBS at different concentration was added and incubated for 1 h at 37°C. The plates were washed 4 times with water, before peroxidase-conjugated F(ab’)_2_ Fragment Donkey anti-Mouse IgM or Goat anti-Mouse IgA (Thermo Fisher, eBioscience) detection antibody (0.5 µg/ml in TBST + 1 mM EDTA) was added and incubated at 37°C for 1 h. Subsequently, the plates were washed 4 times with water, before Super AquaBlue ELISA substrate (Thermo Fisher) was added. The absorbance at 405 nm was measured after 20 min and 90 min on an ELISA plate reader (Biotek). Serum from *Fcmr^-/-^* mice (diluted to 0.5 µg/ml IgM or IgA antibody concentration) served as a positive control. Serum from *Rag2^-/-^* mice was used as a negative control.

### Statistical analysis

Data are presented as mean values ± standard error of the mean (SEM). Statistical analysis was performed using GraphPad Prism (v6.0h, Mac OS X). Unless stated otherwise, differences between means were assessed using one-way ANOVA and Tukey’s multiple comparisons test. A *p* value of <0.05 was considered statistically significant; *p < 0.05, **p < 0.01, ***p < 0.001.

## Data Availability

The raw data supporting the conclusions of this article will be made available by the authors, without undue reservation.

## Supplementary material

Supplementary Figure 1 High capacity of innate B cells to differentiate into IgA plasma cells upon BCR-independent stimulation via TLR4. FACS-sorted B cell subsets (FoB, MZB, and B1a cells) from the spleen of Blimp1-YPF reporter mice were stimulated with **(A, B)** LPS and **(C)** FoBs were stimulated with anti-IgM + anti-CD40 + IL-4. Cells were analyzed on the indicated days for the formation of Blimp1(YFP)^+^IgA^+^ plasma cells or Blimp1(YFP)^+^IgG^+^ plasma cells. Right panels show staining with an isotype matched control antibody.

Supplementary Figure 2 Flow cytometric gating strategy for the identification of donor-derived B-lineage cells from spleen, mesenteric lymph nodes and small intestine. Related to **Figures 3**, **4**. **(A-C)** Representative flow cytometric gating strategy to identify donor-derived B-lineage cells in the indicated organs 3 weeks after adoptive transfer of purified naïve B cell subsets into *Rag2* ^-/-^ host mice. Cells were gated on FSC/SSC, live/dead stain, CD45^+^, singlet cells and donor-derived B lineage cells were identified by staining for CD79b versus a ‘dump’ channel. **(A)** spleen; **(B)** mesenteric lymph nodes; **(C)** small intestine lamina propria.

Supplementary Figure 3 Splenic B1a B cells do not transdifferentiate into MZB-like B cells under lymphopenic conditions. **(A)** B1a B cells and **(B)** FoBs were purified by fluorescence-activated cell sorting and adoptively transferred into *Rag2^-/-^* mice. 3 weeks after cell transfer, spleens of recipient mice were subjected to flow cytometric analysis. FACS plots are gated on live CD45^+^CD19^+^ singlet naïve B cells and show staining for CD1d vs CD23 on input B1a B cells (sorted cells before transfer) and on splenic B cells at week 3 after transfer into *Rag2^-/-^* mice. **(B)** Positive control showing FoBs that have transdifferentiated to CD1d^high^CD23^low^ MZB-like B cells under lymphopenic conditions (3 weeks after transfer of FoBs into *Rag2^-/-^* mice). Numbers indicate the frequency of cells with a CD1d^high^CD23^low^ marginal zone B cell-like phenotype.

Supplementary Figure 4 Serum IgG levels upon adoptive transfer of naïve B cell subsets into lymphopenic *Rag2^-/-^* mice. Follicular B cells (FoB), marginal zone B cells (MZB), and B1a B cells were purified by fluorescence-activated cell sorting and adoptively transferred into *Rag2^-/-^* mice. IgG antibody levels in the serum of *Rag2^-/-^* mice that had received the indicated B cell subsets or from *Rag2^-/-^* control mice in comparison to serum from normal wild type (WT) mice. Each symbol represents an individual mouse; lines indicate mean ± SEM; *nd*, not detectable.
