# Supplementary figures and images for: Acquisition of innate B cell properties and generation of autoreactive IgA antibodies by follicular B cells during homeostatic proliferation
Source: Front Immunol. 2025 Jan 22;16:1506628. doi: 10.3389/fimmu.2025.1506628 (PMC11794109; doi:10.3389/fimmu.2025.1506628)

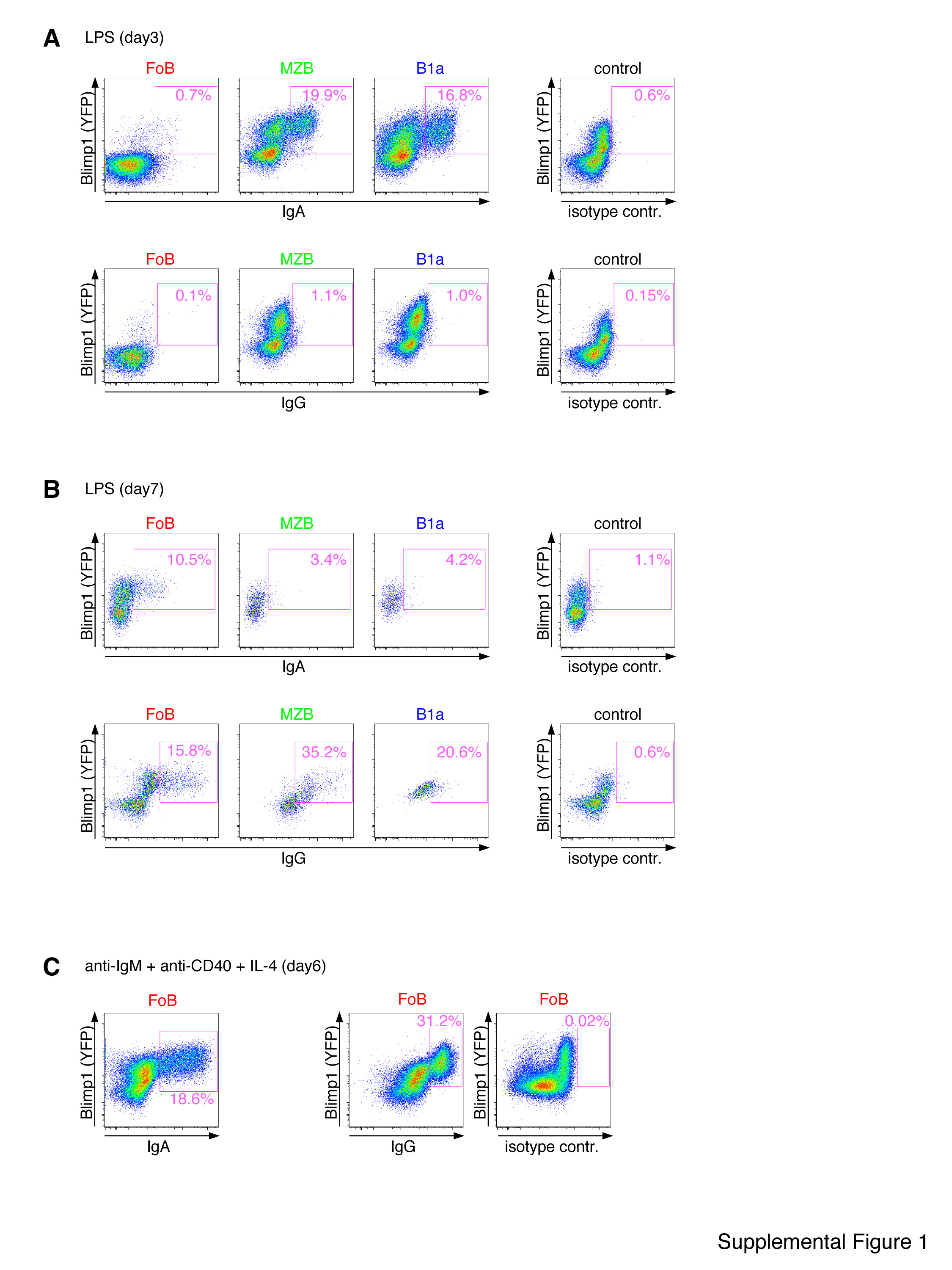

Supplement: Supplementary Figure 1 — High capacity of innate B cells to differentiate into IgA plasma cells upon BCR-independent stimulation via TLR4. FACS-sorted B cell subsets (FoB, MZB, and B1a cells) from the spleen of Blimp1-YPF reporter mice were stimulated with (A, B) LPS and (C) FoBs were stimulated with anti-IgM + anti-CD40 + IL-4. Cells were analyzed on the indicated days for the formation of Blimp1(YFP)+IgA+ plasma cells or Blimp1(YFP)+IgG+ plasma cells. Right panels show staining with an isotype matched control antibody. [file Image1.jpg]

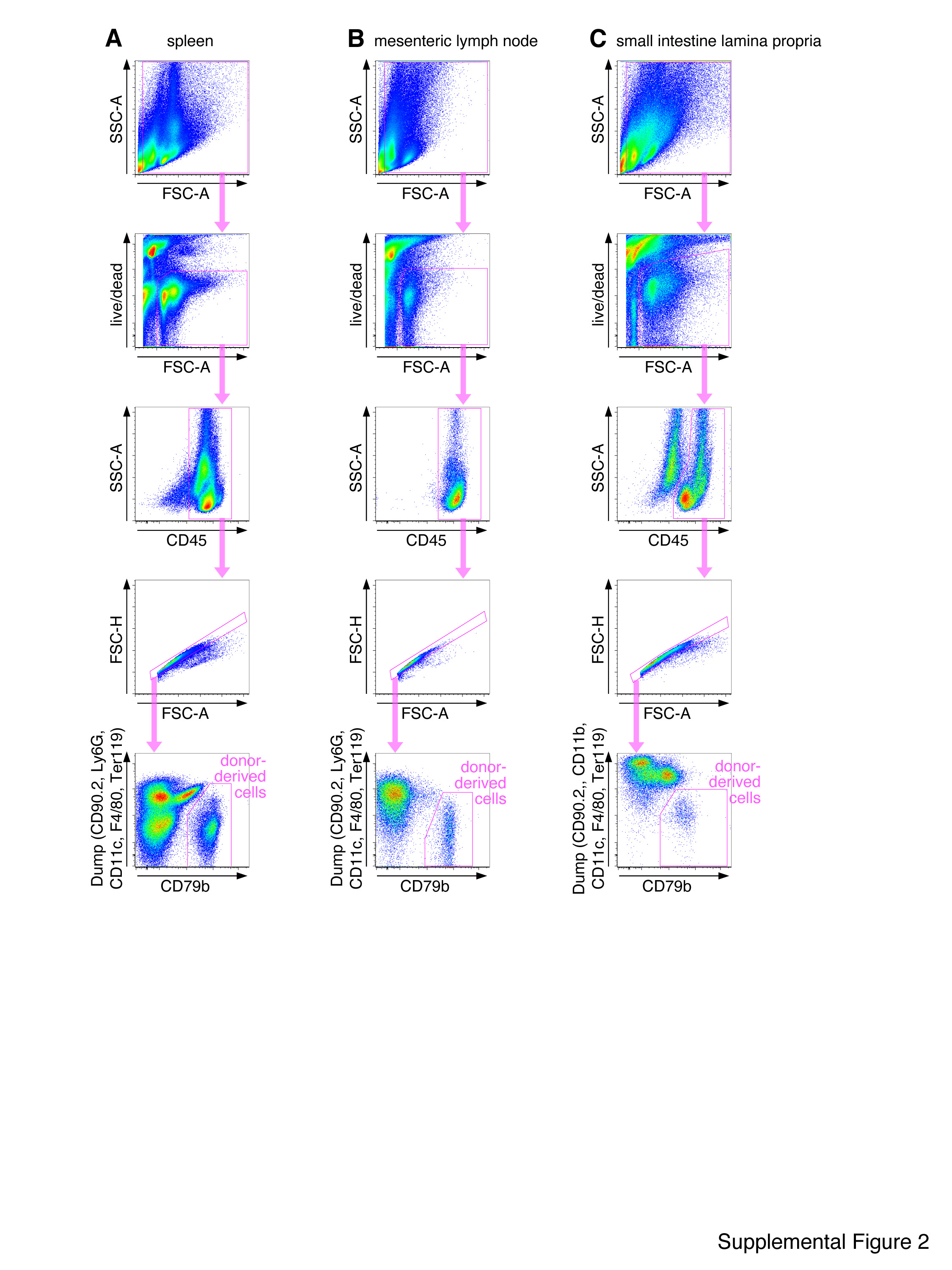

Supplement: Supplementary Figure 2 — Flow cytometric gating strategy for the identification of donor-derived B-lineage cells from spleen, mesenteric lymph nodes and small intestine. Related to Figures 3 , 4 . (A-C) Representative flow cytometric gating strategy to identify donor-derived B-lineage cells in the indicated organs 3 weeks after adoptive transfer of purified naïve B cell subsets into Rag2 -/- host mice. Cells were gated on FSC/SSC, live/dead stain, CD45+, singlet cells and donor-derived B lineage cells were identified by staining for CD79b versus a ‘dump’ channel. (A) spleen; (B) mesenteric lymph nodes; (C) small intestine lamina propria. [file Image2.jpeg]

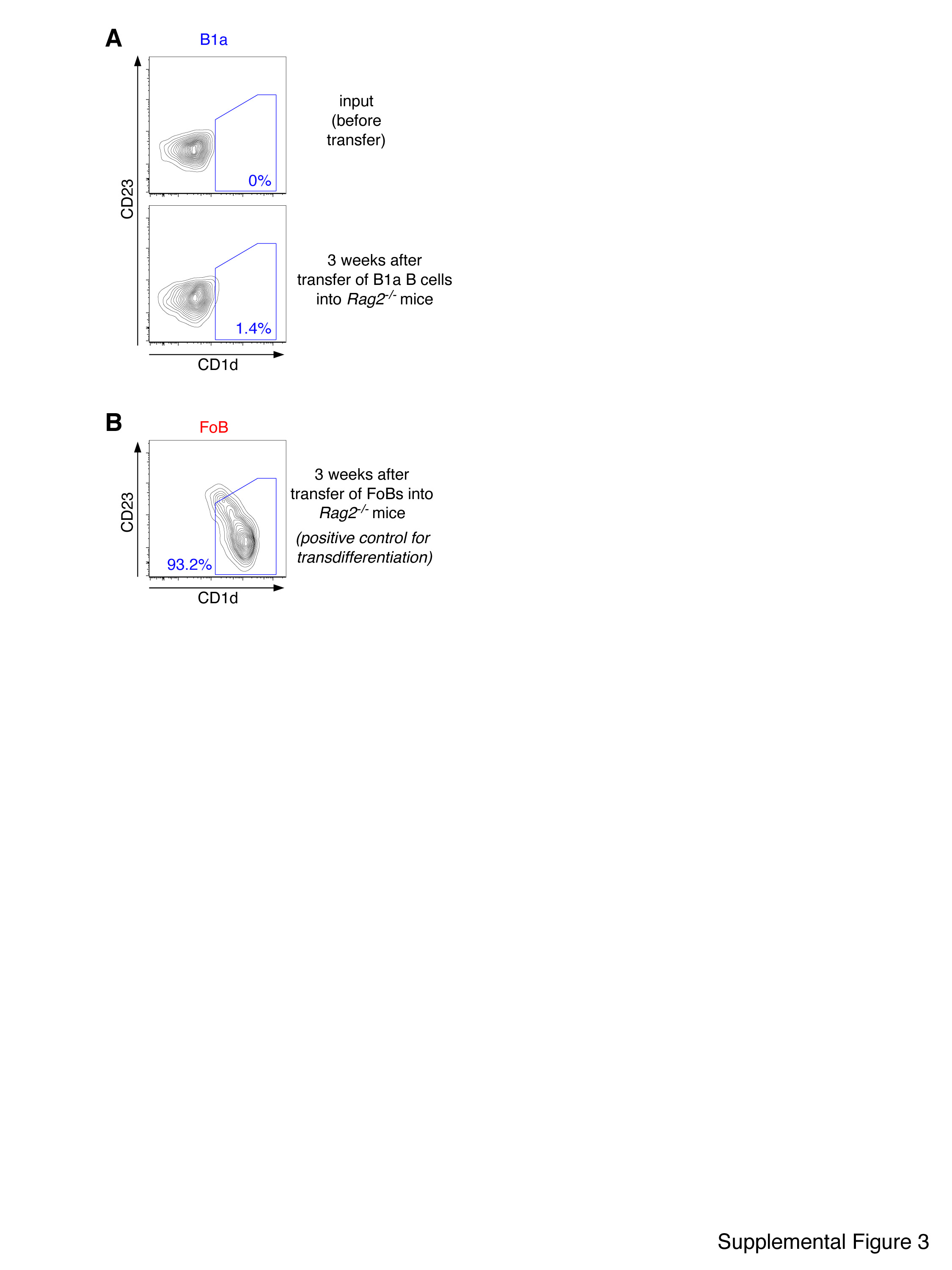

Supplement: Supplementary Figure 3 — Splenic B1a B cells do not transdifferentiate into MZB-like B cells under lymphopenic conditions. (A) B1a B cells and (B) FoBs were purified by fluorescence-activated cell sorting and adoptively transferred into Rag2-/- mice. 3 weeks after cell transfer, spleens of recipient mice were subjected to flow cytometric analysis. FACS plots are gated on live CD45+CD19+ singlet naïve B cells and show staining for CD1d vs CD23 on input B1a B cells (sorted cells before transfer) and on splenic B cells at week 3 after transfer into Rag2-/- mice. (B) Positive control showing FoBs that have transdifferentiated to CD1dhighCD23low MZB-like B cells under lymphopenic conditions (3 weeks after transfer of FoBs into Rag2-/- mice). Numbers indicate the frequency of cells with a CD1dhighCD23low marginal zone B cell-like phenotype. [file Image3.jpeg]

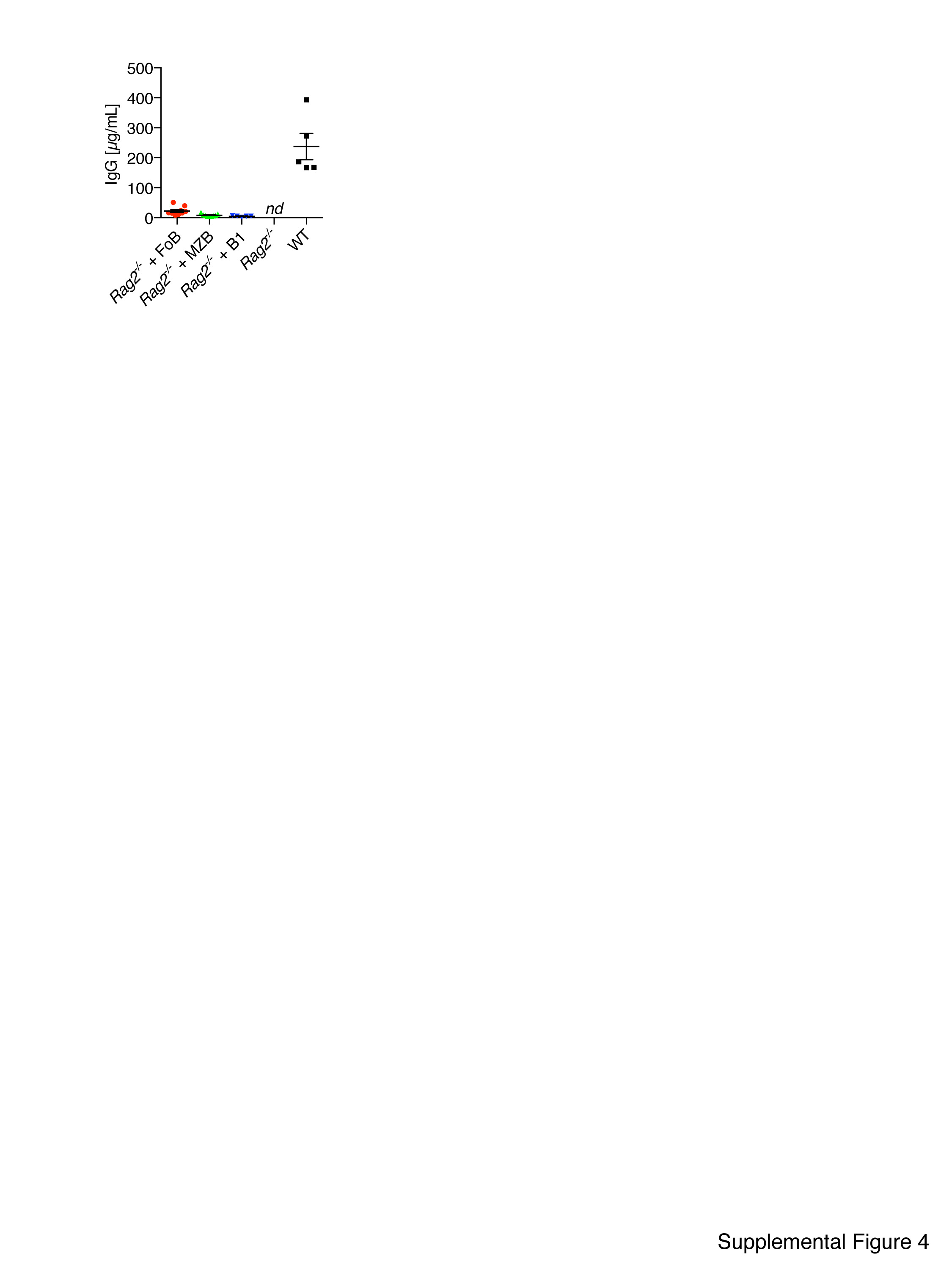

Supplement: Supplementary Figure 4 — Serum IgG levels upon adoptive transfer of naïve B cell subsets into lymphopenic Rag2-/- mice. Follicular B cells (FoB), marginal zone B cells (MZB), and B1a B cells were purified by fluorescence-activated cell sorting and adoptively transferred into Rag2-/- mice. IgG antibody levels in the serum of Rag2-/- mice that had received the indicated B cell subsets or from Rag2-/- control mice in comparison to serum from normal wild type (WT) mice. Each symbol represents an individual mouse; lines indicate mean ± SEM; nd, not detectable. [file Image4.jpeg]
